# Multirecombinant Enterovirus A71 Subgenogroup C1 Isolates Associated with Neurologic Disease, France, 2016–2017

**DOI:** 10.3201/eid2506.181460

**Published:** 2019-06

**Authors:** Stéphanie Tomba Ngangas, Alexander Lukashev, Gwendoline Jugie, Olga Ivanova, Jean-Michel Mansuy, Catherine Mengelle, Jacques Izopet, Anne-Sophie L’honneur, Flore Rozenberg, David Leyssene, Denise Hecquet, Stéphanie Marque-Juillet, David Boutolleau, Sonia Burrel, Hélène Peigue-Lafeuille, Christine Archimbaud, Kimberley Benschop, Cécile Henquell, Audrey Mirand, Jean-Luc Bailly

**Affiliations:** Université Clermont Auvergne, Clermont-Ferrand, France (S. Tomba Ngangas, G. Jugie, H. Peigue-Lafeuille, C. Archimbaud, C. Henquell, A. Mirand, J.-L. Bailly);; Sechenov University, Moscow, Russia (A. Lukashev);; Chumakov Federal Scientific Center for Research and Development of Immune-and-Biological Products, Moscow (O. Ivanova);; Centre Hospitalier Universitaire de Toulouse, Toulouse, France (J.-M. Mansuy, C. Mengelle, J. Izopet);; Assistance Publique-Hôspitaux de Paris Cochin, Paris, France (A.-S. L’honneur, F. Rozenberg);; Centre Hospitalier de la Côte Basque, Bayonne, France (D. Leyssene);; Centre Hospitalier Universitaire Amiens, Amiens, France (D. Hecquet);; Centre Hospitalier de Versailles, Le Chesnay, France (S. Marque-Juillet);; Assistance Publique-Hôspitaux de Paris Pitié-Salpêtrière-Charles Foix, Paris (D. Boutolleau, S. Burrel);; CHU Clermont-Ferrand, Clermont-Ferrand (H. Peigue-Lafeuille, C. Archimbaud, C. Henquell, A. Mirand, J.-L. Bailly);; National Institute for Public Health and the Environment, Bilthoven, the Netherlands (K. Benschop)

**Keywords:** epidemiologic monitoring, whole-genome sequencing, genetic recombination, neurologic manifestations, enterovirus infection, enterovirus, viruses, neurologic disease, France, EV-A71, children, C1 subgenogroup, most recent common ancestor, coxsackievirus, C1v2015 lineage, genomic region P1, 3Dpol, 5′ UTR

## Abstract

In 2016, an upsurge of neurologic disease associated with infection with multirecombinant enterovirus A71 subgenogroup C1 lineage viruses was reported in France. These viruses emerged in the 2000s; 1 recombinant is widespread. This virus lineage has the potential to be associated with a long-term risk for severe disease among children.

Enterovirus A71 (EV-A71) comprises 7 genogroups (A–G) and various subgenogroups (e.g., B0–B5, C1–C5) (*1*). B4, B5, and C4 viruses circulate mainly in Asia, and C1 and C2 viruses have been detected in Europe (*2*). In 2016, an upsurge in neurologic manifestations of enterovirus infection was reported in France (*3*). These cases were associated with an emerging lineage of subgenogroup C1 enteroviruses first reported in 2015 in Germany and later in Spain and 4 other countries (Figure 1, panel A) (*4*–*8*). Our aim was to obtain the full genomes of the viruses from the specimens collected in France and track down the origin of this emerging lineage, hereafter referred to as C1v2015.

**Figure 1:**
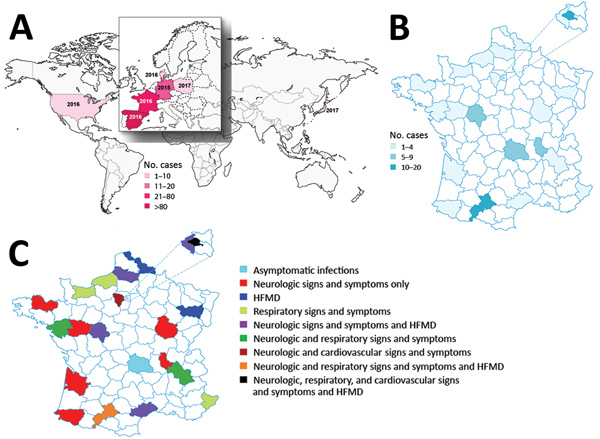
Geographic locations and numbers of enterovirus A71 (EV-A71) subgenogroup C1v2015 infections reported during 2015–2017. A) Countries in which EV-A71 C1v2015 was reported. The year the virus was first reported is indicated. The size of Europe is increased for easier visualization. B) Geographic distribution and number of cases of EV-A71 C1v2015 infection reported in hospitals, by department, France, 2016–2017. C) Geographic distribution of clinical manifestations associated with EV-A71 C1v2015 infection reported in hospitals, by department, France, 2016–2017. The size of a select set of departments is enlarged for easier visualization. HFMD, hand, foot and mouth disease.

## The Study

According to consolidated data recorded from the French Enterovirus Surveillance Network, 77 laboratory-confirmed cases of C1v2015 infection occurred during March–October 2016; in comparison, 136 EV-A71 infections of all genogroups combined were recorded during 2010–2015. The C1v2015 cases were widespread throughout France and associated with various clinical manifestations, including meningitis, cerebellitis, encephalitis, and myelitis, as well as hand, foot and mouth disease (HFMD) (Figure 1, panels B, C). One fatal case resulted from HFMD and cardiorespiratory failure. We analyzed 32 clinical specimens available from 25 patients reported as having a C1v2015 infection in 2016 and 2017 (Table 1; Appendix Table 1). Specimens and clinical data were collected during routine clinical work-up and epidemiologic surveillance, and patient data were deidentified before this study was conducted. The study was approved by the review board Comité de Protection des Personnes Sud-Est VI (no. 2018/CE44) in Clermont-Ferrand, France. The study population comprised 16 hospitalized children (median age 0.1 years), 4 children seen via ambulatory care (median age 1.8 years), and 5 children with asymptomatic infection (median age 1.4 years) in a childcare facility placed under community surveillance. We obtained the complete genomes, including the full 5′ and 3′ untranslated regions (UTRs), of 18 of 20 specimens and partial genomes of 2 of 20 specimens (2,893-nt and 4,380-nt long) acquired from 18 children (Appendix) (*2*). We also determined the genomes of 12 isolates recovered during routine enterovirus surveillance to investigate their genetic relationships with C1v2015 (Appendix Table 2); we selected these viruses on the basis of previous exploratory investigations of their partial sequences (*2*,*9*,*10*).

**Table 1:** Characteristics of patients with EV-A71 subgenogroup C1v2015 infection, France, 2016–2017*

Patient no.	Specimen no.	Care setting (City)	Clinical diagnosis	Specimen material	Collection date	C_t_
01	01†	Hospital (Toulouse)	Acute meningitis	Throat swab	2016 May 3	25
02	02	Hospital (Toulouse)	Fever	Nasopharyngeal aspirate	2016 May 19	30
02	03	Hospital (Toulouse)	Fever	Feces	2016 May 19	31
03	04	Hospital (Paris)‡	Fever	Plasma	2016 Jun 7	37
04	05	Hospital (Paris)‡	Fever	Plasma	2016 Jun 10	32
05	06	Hospital (Bayonne)	Encephalitis	Cerebrospinal fluid	2016 Jun 12	35
05	07†	Hospital (Bayonne)	Encephalitis	Throat swab	2016 Jun 24	35
05	08	Hospital (Bayonne)	Encephalitis	Rectal swab	2016 Jun 24	35
06	09	Hospital (Toulouse)	Infant fever	Feces	2016 Jul 10	31
07	10†	Hospital (Toulouse)	Sepsis-like disease	Throat swab	2016 Aug 10	24
07	11†	Hospital (Toulouse)	Sepsis-like disease	Nasopharyngeal aspirate	2016 Aug 10	NR
08	12†	Hospital (Paris)‡	Convulsions	Nasopharyngeal aspirate	2016 Aug 11	28
09	13§	Ambulatory (Mirecourt)	HFMD	Mouth swab	2016 Aug 30	32
10	14†	Ambulatory (Mirecourt)	HFMD	Throat swab	2016 Aug 30	29
11	15†	Hospital (Paris)‡	Fever, hypotonia	Blood	2016 Sep 5	30
12	16§	Hospital (Toulouse)	Acute meningitis, HFMD	Throat swab	2016 Sep 7	33
13	17†	Ambulatory (Toulouse)	HFMD	Mouth swab	2016 Sep 14	29
14	18†	Hospital (Paris)‡	Fever	Feces	2016 Sep 27	20
15¶	19†	Daycare (Volvic)	NR	Feces	2016 Oct 4	29
16¶	20†	Daycare (Volvic)	NR	Feces	2016 Oct 4	31
17¶	21†	Daycare (Volvic)	NR	Feces	2016 Oct 4	31
18¶	22†	Daycare (Volvic)	NR	Feces	2016 Oct 4	29
19¶	23†	Daycare (Volvic)	NR	Feces	2016 Oct 4	31
20	24	Hospital (Toulouse)	Sepsis-like disease	Throat swab	2016 Oct 5	33
21	25	Hospital (Versailles)	Diarrhea	Cerebrospinal fluid	2016 Oct 9	35
22	26	Hospital (Toulouse)	Acute meningitis, cerebellitis	Throat swab	2016 Oct 10	36
22	27	Hospital (Toulouse)	Acute meningitis, cerebellitis	Feces	2016 Oct 10	30
23	28†	Hospital (Toulouse)	Fever	Throat swab	2016 Oct 11	27
23	29	Hospital (Toulouse)	Fever	Feces	2016 Oct 12	29
24	30†	Hospital (Amiens)	Myelitis	Nasopharyngeal swab	2016 Oct 18	30
24	31†	Hospital (Amiens)	Myelitis	Feces	2016 Oct 20	33
25	32†	Ambulatory (Montesson)	Atypical HFMD, herpangina	Throat swab	2017 Jul 3	22

*See Appendix Table 1 (https://wwwnc.cdc.gov/EID/article/25/6/18-1460-App1.pdf) for extended data, including GenBank accession nos. C_t_, cycle threshold; EV-A71, enterovirus A71; HFMD, hand, foot and mouth disease; NR, not reported. †Specimens for which the complete viral genomes (including the full 5′ and 3′ untranslated regions) were obtained. ‡Assistance Publique-Hôpitaux de Paris Cochin, Paris, France. §Specimens for which partial genomes were obtained. ¶Patients 15-19 were children in the same daycare facility who had no evidence of clinical disease.

**Figure 2:**
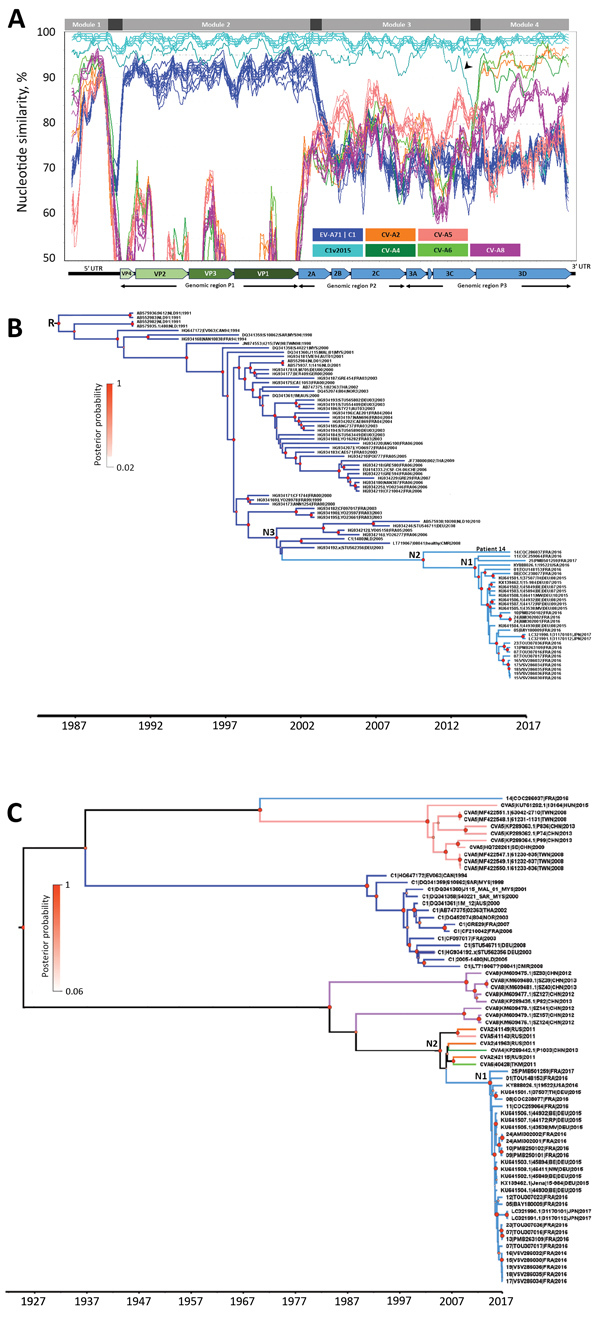
Nucleotide similarity and phylogenetic analyses of EV-A71 subgenogroup C1v2015 isolates, France, 2016–2017, constructed to determine temporal origin of C1v2015 lineage. A) Nucleotide similarity patterns between EV-A71 C1v2015 and other EV-A lineages indicate the C1v2015 genome has a mosaic structure. The genome of the virus from patient 10’s throat swab (10|PMB250102|FRA|2016) was used as the query sequence. The similarity plots determined with the other C1v2015 genomes (except 14|COC286037|FRA|2016) were similar. A schematic diagram of the enterovirus genome is shown at the bottom of the panel. Nucleotide similarity was calculated by the sliding window method (window of 300 nt moving every 30 nt). Four genomic modules (labeled at top of panel) with different genetic origins are identified. The 99% CIs of the nucleotide boundaries assessed for the genomic modules (indicated in dark gray) were determined as described in Hassel et al. (*11*). The 3′ end of module 1 and 5′ end of module 2 were located at the end of the 5′ UTR but were not determined precisely. The 3′ end of module 2 was located between nucleotides 3,532 and 3,722. The 5′ end of module 4 was located at the end of the 3Cpro gene (nucleotides 5,968–6,044). The arrowhead indicates a previously undescribed recombinant lineage of C1v2015 (Appendix Figure 2, https://wwwnc.cdc.gov/EID/article/25/6/18-1460-App1.pdf). B) Phylogenetic tree constructed by using genomic region P1, encoding capsid proteins VP1–VP4, and methods described earlier (*11*). We performed this analysis with 85 sequences assigned to the EV-A71 C1 and C1v2015 lineages. Tree shows the temporal distribution of lineages, including the emergence of lineage C1v2015. The sequences used as references were labeled with GenBank accession numbers. C) Phylogenetic tree constructed by using the 3Dpol gene encoding the viral RNA polymerase common to C1v2015 and several CV-A strains. The dataset comprised 70 sequences: 24 CV-A (including 5 sequences from this study), 14 EV-A71 C1 (including 6 sequences from this study), 12 publicly available C1v2015, and 20 C1v2015 sequences from this study. Recombination analyses provided no evidence of internal breakpoints within the sequences. N1 represents the time to most recent common ancestor (MRCA) of all included EV-A71 C1v2015 isolates except the virus from patient 14; N2 in panel B represents the MRCA of all EV-A71 C1v2015 isolates, including the virus from patient 14; N2 in panel C represents the MRCA of EV-A71 C1v2015 and its parent C1 lineage; and N3 represents the MRCA of EV-A71 C1v2015 and its parent C1 lineage. Diameters of circles at nodes reflect posterior probability. Branches of trees are color coded according to virus lineage as indicated in panel A. AUS, Australia; AUT, Austria; CAN, Canada; CHE, Switzerland; CHN, China; CMR, Cameroon; C1v2015, enterovirus subgenogroup C1 strain discovered in 2015; CV-A, coxsackievirus A; DEU, Germany; EV-A71C1, enterovirus A71 subgenogroup C1; FRA, France; JPN, Japan; N, node; NLD, the Netherlands; NOR, Norway; MYS, Malaysia; RUS, Russia; THA, Thailand; TKM, Turkmenistan; TWN, Taiwan; USA, United States; UTR, untranslated region; VP, viral protein.

We performed whole-genome sequence analyses as previously described (*11*) to identify which viruses were the closest relatives of C1v2015. The C1v2015 genome appears to be a mosaic comprising 4 modules defined by distinct patterns of similarity possibly arising through recombination (Figure 2, panel A). The nucleotide similarity patterns for module 2 (genomic region P1 comprising 4 capsid protein genes) suggest this region was inherited en bloc from an earlier subgenogroup C1 lineage. We used genomic region P1 to determine the evolutionary relatedness between C1v2015 and earlier C1 viruses and to date when the upsurge of C1v2015 infections began in Europe (Figure 2, panel B). All C1v2015 viruses clustered in a lineage distinct from that comprising the C1 viruses reported during 1991–2010. The nucleotide substitution rate of C1v2015 (5.2238 [95% highest probability density HPD interval 4.124–6.3737] × 10^–3^ nt substitutions/y) and earlier C1 lineages (4.6302 [95% HPD interval 4.1769–5.1353] × 10^–3^ nt substitutions/y) was similar. All of the P1 sequences from these viruses, except that of the virus from patient 14, had a maximum nucleotide sequence difference from each other of 2%; the P1 sequence of the virus from patient 14 differed from that of other C1v2015 viruses by 4.8%. The close genetic relatedness between the C1v2015 sequences reported during 2015–2017 in France, Germany, Japan, and the United States was indicative of rapid widespread transmission. We estimated that interpersonal transmission of this lineage began during 2009–2011 (Table 2; Figure 2, panel B) and that its spread was sustained during 2013–2014, just 1–2 years before C1v2015 was first reported. The most recent common ancestor between C1v2015 and earlier C1 viruses was dated to 2000–2002. Seven EV-A71 subgenogroup C1 viruses from Africa and Europe were located at the base of the C1v2015 lineage (Figure 2, panel B), suggesting that the C1 strain involved in the emergence of C1v2015 was circulating in this region during the 2000s.

**Table 2:** Estimation of year of MRCA of EV-A71 subgenogroup C1v2015 lineage by using different enteroviruses*

Node†	Year of MRCA (95% HPD interval)	
	Genomic region P1	3Dpol gene
1‡	2013.6 (2013.2–2014.1)	2013.6 (2012.9–2014.3)
2§	2010.2 (2009–2011.3)	2004.1 (2001.7–2006.2)
3¶	2000.5 (2000.1–2001.6)	ND
Root	1986 (1984.7–1987.3)	ND

*EV-A71, enterovirus A71; HPD, highest probability density; MRCA, most recent common ancestor; ND, not done. †MRCAs were determined for nodes and root in Figure 2. ‡Node 1 represents the MRCA of all included EV-A71 C1v2015 isolates except the virus from patient 14. §For genomic region P1, node 2 represents the MRCA of all EV-A71 C1v2015 isolates, including the virus from patient 14. For 3Dpol gene, node 2 represents the MRCA of EV-A71 C1v2015 and its parent C1 lineage. ¶Node 3 represents the MRCA of EV-A71 C1v2015 and its parent C1 lineage.

The C1v2015 genomic module 4 comprises the entire 3Dpol gene and has a 90%–95% nucleotide similarity with 4 distinct EV-A genomes: coxsackievirus A2 (CV-A2) and CV-A5 from Russia, CV-A4 from China, and CV-A6 from Turkmenistan (Figure 2, panel A). We performed another phylogenetic analysis to assess the temporal origin of C1v2015 using this module. With the 3Dpol phylogenetic analysis, we estimated that C1v2015 began spreading in 2010–2014 (Table 2; Figure 2, panel C), an estimate similar to that calculated with the P1 phylogeny. The nucleotide substitution rates with this analysis were also similar (C1v2015 3.7689 [95% HPD interval 1.3003–6.5838] × 10^–3^ nt substitutions/y and C1 3.6318 [95% HPD interval 1.6064–6.2072] × 10^–3^ nt substitutions/y). Whole-genome sequencing analysis showed that the isolate from patient 14 (14|COC286037|FRA|2016) shared distinct 3Dpol genes with other C1v2015 viruses (Appendix Figure 1). Overall, data indicate that the virus from patient 14 was an early recombinant of the C1v2015 lineage (Appendix Figure 2).

Within genomic module 1 (5′ UTR, first 600 nt), we found areas of moderate nucleotide similarity (90%–95%) between the C1v2015 genome and the CV-A6 and CV-A8 genomes and lower similarity (<88%) with the EV-A71 subgenogroup C1 genomes (Figure 2, panel A). The C1v2015 5′ UTR was therefore inherited from an EV-A lineage virus but not from the C1 ancestors that provided the capsid region. The pattern of sequence variation in the 5′ UTR precludes the possibility of analysis with a molecular clock.

The genomic module 3 of C1v2015 had low similarity with all the publicly available EV-A genomes; thus, the precise origin remains unknown (Figure 2, panel A). The highest nucleotide similarity scores (<90% with CV-A5 genomes) indicate only a distant genetic relationship. We conclude that genes 2A (except the 5′ terminus), 2B, 2C, and 3A–3C were transferred into the C1v2015 genome from a previously unreported lineage.

## Conclusions

Thirty years after the outbreaks in central Europe (*12*,*13*), the 2016 upsurge of infections is a reminder that EV-A71 is of growing public health concern. After the B5 and C4 subgenogroup upsurges, C1v2015 is the latest example of an emerging recombinant EV-A71 associated with neurologic manifestations. Recombination, which frequently occurs in enteroviruses, is considered a factor driving this viral emergence (*14*,*15*). Compared with earlier circulating lineages of EV-A71, C1v2015 is a multirecombinant that arose through complete shuffling of all nonstructural genomic regions, although the capsid genes are phylogenetically typical of C1 viruses. Shuffling involved >2 recombination events with EV-A genomes before the emergence of C1v2015 as a life-threatening pathogen (Appendix Figure 2). From a public health perspective, the spread of C1v2015 could have resulted from acquired genomic features, notably a unique combination of the 5′ UTR and 3Dpol gene, because recombination events clearly preceded the extensive circulation of C1v2015. The mosaic structure of the genome indicates that C1v2015 is an integral part of a large recombination network including multiple EV-A viruses transmitted in Eurasia. Given the propensity of enteroviruses to recombine their genomes and spread rapidly across distant countries (*2*,*11*) and that C1v2015 circulation continued throughout 2017 and 2018 in France, we need to determine if this virus is associated with a long-term recurrent risk for severe disease in the pediatric population through sharing data from global surveillance.

**Appendix.** Additional information in study of enterovirus A71 subgenogroup C1 isolates associated with neurologic disease, France, 2016–2017.
