# Correction: Vol. 25, No. 6

**DOI:** 10.3201/eid2508.C22508

**Published:** 2019-08

**Keywords:** errata, erratum, correction

Author Olga Ivanov should have also been listed as affiliated with Sechenov University, Moscow, Russia, in Multirecombinant Enterovirus A71 Subgenogroup C1 Isolates Associated with Neurologic Disease, France, 2016–2017 (S. Tomba Ngangas et al.). The article has been corrected online (https://wwwnc.cdc.gov/eid/article/25/6/18-1460_article).

