# Supplementary material for: Multirecombinant Enterovirus A71 Subgenogroup C1 Isolates Associated with Neurologic Disease, France, 2016–2017
Source: Emerg Infect Dis. 2019 Jun;25(6):1204–8. doi: 10.3201/eid2506.181460 (PMC6537711; doi:10.3201/eid2506.181460)
Supplement: Supplementary file 1 — Appendix. Additional information in study of enterovirus A71 subgenogroup C1 isolates associated with neurologic disease, France, 2016–2017. [file 18-1460-Techapp-s1.pdf]

Article DOI: https://doi.org/10.3201/eid2506.181460

Multirecombinant Enterovirus A71 Subgenogroup C1 Isolates Associated with Neurologic Disease, France, 2016–2017

Appendix

Materials and Methods

Amplification of Gene Sequences and Nucleotide Sequencing of PCR Products

Viral RNA was extracted from patient specimens or cell culture supernatants with the NucliSENS EasyMAG semi-automatic extractor (BioMérieux, https://www.biomerieux.com) using specific B protocol. The complete genomes were amplified into 2 amplicons (A, 5′ untranslated region [UTR] – 2C; no. 4,500 bp) and (B, 2C – 3′ UTR; no. 3,000 bp). cDNA synthesis was performed with SuperScript III reverse transcription (Invitrogen, https://www.thermofisher.com/us/en/home/brands/invitrogen.html) using a single primer located within the 2C and the 3′ UTR. PCR was performed with Invitrogen Platinum SuperFi PCR Master Mix in 20 µL total volume with 2 µL of cDNA, 10 µL of PCR Master Mix, 6 µL of H_2O, and 1 µL of each primer (Appendix Table 3). PCR primers are indicated for both amplicons in Appendix Table 3. Gene amplification reactions to obtain the 5′ UTR-2C amplicon were performed under the following conditions: 98°C for 30 s, followed by 41 cycles of 98°C for 5 s, 66°C for 10 s, and 72°C for 2 min 20 s, with a final extension step at 72°C for 5 min. To obtain the 2C-3′ UTR amplicon, the reaction conditions were 98°C for 30 s, followed by 41 cycles of 98°C for 5 s, 70°C for 10 s, and 72°C for 2 min 25 s, with a final extension step at 72°C for 5 min. PCR products were visualized by 1% agarose gel electrophoresis. Sequencing was performed as previously described (*1*) with the Big Dye Cycle Sequencing Kit version 3.0 and an ABI 3500Dx automated DNA sequencer (Applied Biosystems, https://www.thermofisher.com/us/en/home/brands/applied-biosystems.html).

Bayesian Coalescent Analyzes

Two parameters, evolutionary rate and divergence times, were inferred from the P1 genomic region encoding the 4 capsid proteins and 3D polymerase gene of enterovirus A71 (EV-A71) and EV-A. The sequence data sets were analyzed with a Bayesian Markov chain Monte Carlo algorithm allowing estimation of the posterior distribution of parameters, which is implemented in the BEAST v1.8.4 program (http://beast.community) (*2*). The nucleotide substitution model used was the general time reversible model with 4 gamma rate categories, invariant sites, and partition of the 3 codon positions. A relaxed molecular clock assuming uncorrelated lognormal prior distributions of substitution rates among lineages was used with the Bayesian skyline, which does not assume a specified model of demography (*3*,*4*). The analyzes were run for 50 million generations, sampling a tree every 5,000 steps and discarding the first 10% as burn-in. Markov chain Monte Carlo convergence and effective sample sizes were checked using the TRACER v1.6 program (http://tree.bio.ed.ac.uk/software/tracer). Analyses were considered to have converged and reached stability after the burn-in period when effective sample sizes were >200. Uncertainty in the estimates was indicated by the 95% highest probability density values. The TreeAnnotator v1.5.4 program (http://beast.community/) computed the maximum clade credibility tree from all plausible trees created during the BEAST run, with the first 10% of trees removed as burn-in. The tree was annotated by using FigTree v1.4.3 (http://tree.bio.ed.ac.uk/software/figtree).

Recombination Detection in Complete Genomes

The EV-A71 C1v2015 genome sequences were compared with 12 EV-A determined in the present study and 31 genomes selected among those available in international databases. The nucleotide similarity patterns were determined with the SimPlot v3.5.1 program (*5*) with a sliding window of 200 nt moving in steps of 20 nt. Other methods implemented in RDP v.4.33 (*6*) (http://web.cbio.uct.ac.za/~darren/rdp.html) were used to locate the positions of possible recombination events. The default methods, RDP (*7*), Geneconv (*8*), MaxChi (*9*), Chimaera (*10*), Bootscan (*11*), and SiScan (*12*) were used to survey the sequences. Only putative recombinant signals detected with 5 methods were considered to exclude the possibility of false-positive detection.

References

1. Hassel C, Mirand A, Farkas A, Diedrich S, Huemer HP, Peigue-Lafeuille H, et al.; HFMD French Study Network. Phylogeography of coxsackievirus A16 reveals global transmission pathways and recent emergence and spread of a recombinant genogroup. J Virol. 2017;91:e00630-17. PubMed http://dx.doi.org/10.1128/JVI.00630-17

2. Drummond AJ, Rambaut A. BEAST: Bayesian evolutionary analysis by sampling trees. BMC Evol Biol. 2007;7:214. PubMed http://dx.doi.org/10.1186/1471-2148-7-214

3. Drummond AJ, Rambaut A, Shapiro B, Pybus OG. Bayesian coalescent inference of past population dynamics from molecular sequences. Mol Biol Evol. 2005;22:1185–92. PubMed http://dx.doi.org/10.1093/molbev/msi103

4. Drummond AJ, Ho SYW, Phillips MJ, Rambaut A. Relaxed phylogenetics and dating with confidence. PLoS Biol. 2006;4:e88. PubMed http://dx.doi.org/10.1371/journal.pbio.0040088

5. Lole KS, Bollinger RC, Paranjape RS, Gadkari D, Kulkarni SS, Novak NG, et al. Full-length human immunodeficiency virus type 1 genomes from subtype C-infected seroconverters in India, with evidence of intersubtype recombination. J Virol. 1999;73:152–60. PubMed

6. Heath L, van der Walt E, Varsani A, Martin DP. Recombination patterns in aphthoviruses mirror those found in other picornaviruses. J Virol. 2006;80:11827–32. PubMed http://dx.doi.org/10.1128/JVI.01100-06

7. Martin D, Rybicki E. RDP: detection of recombination amongst aligned sequences. Bioinformatics. 2000;16:562–3. PubMed http://dx.doi.org/10.1093/bioinformatics/16.6.562

8. Padidam M, Sawyer S, Fauquet CM. Possible emergence of new geminiviruses by frequent recombination. Virology. 1999;265:218–25. PubMed http://dx.doi.org/10.1006/viro.1999.0056

9. Smith JM. Analyzing the mosaic structure of genes. J Mol Evol. 1992;34:126–9. PubMed http://dx.doi.org/10.1007/BF00182389

10. Posada D, Crandall KA. Evaluation of methods for detecting recombination from DNA sequences: computer simulations. Proc Natl Acad Sci U S A. 2001;98:13757–62. PubMed http://dx.doi.org/10.1073/pnas.241370698

11. Martin DP, Posada D, Crandall KA, Williamson C. A modified bootscan algorithm for automated identification of recombinant sequences and recombination breakpoints. AIDS Res Hum Retroviruses. 2005;21:98–102. PubMed http://dx.doi.org/10.1089/aid.2005.21.98

12. Gibbs MJ, Armstrong JS, Gibbs AJ. Sister-scanning: a Monte Carlo procedure for assessing signals in recombinant sequences. Bioinformatics. 2000;16:573–82. PubMed http://dx.doi.org/10.1093/bioinformatics/16.7.573

Table 1. Characteristics of patients with EV-A71 C1v2015 infection included in the study*

Patient no.†	Specimen no.	Age, y/sex	Care setting (City)	Clinical diagnosis	Specimen material	Collection date	C_t	Sequence designation	GenBank accession no.‡
01	01§	2.6/F	Hospital (Toulouse)	Acute meningitis	Throat swab	2016 May 3	25	01|TOU148153|FRA|2016	LR027527
02	02	0.09/M	Hospital (Toulouse)	Fever	Nasopharyngeal aspirate	2016 May 19	30	02|TOU148165|FRA|2016	
02	03	0.09/M	Hospital (Toulouse)	Fever	Feces	2016 May 19	31	02|TOU148166|FRA|2016	
03	04	0.1/M	Hospital Cochin (Paris)	Fever	Plasma	2016 Jun 7	37	03|COC166142|FRA|2016	
04	05	0.11/F	Hospital Cochin (Paris)	Fever	Plasma	2016 Jun 10	32	04|COC166146|FRA|2016	
05	06	15.6/F	Hospital (Bayonne)	Encephalitis	Cerebrospinal fluid	2016 Jun 12	35	05|BAY180005|FRA|2016	
05	07§	15.6/F	Hospital (Bayonne)	Encephalitis	Throat swab	2016 Jun 24	35	05|BAY180009|FRA|2016	LR027529
05	08	15.6/F	Hospital (Bayonne)	Encephalitis	Rectal swab	2016 Jun 24	35	05|BAY180095|FRA|2016	
06	09	0.08/M	Hospital (Toulouse)	Fever	Feces	2016 Jul 10	31	06|TOU207118|FRA|2016	
07	10§	0.02/M	Hospital (Toulouse)	Sepsis-like disease	Throat swab	2016 Aug 10	24	07|TOU307016|FRA|2016	LR027521
07	11§	0.02/M	Hospital (Toulouse)	Sepsis-like disease	Nasopharyngeal aspirate	2016 Aug 10	NR	07|TOU307017|FRA|2016	LR027522
08	12§	7.6/F	Hospital Cochin (Paris)	Convulsions	Nasopharyngeal aspirate	2016 Aug 11	28	08|COC238077|FRA|2016	LR027528
09	13¶	1.93/F	Ambulatory (Mirecourt)	HFMD	Mouth swab	2016 Aug 30	32	09|PMB250101|FRA|2016	LR027523
10	14§	1.39/M	Ambulatory (Mirecourt)	HFMD	Throat swab	2016 Aug 30	29	10|PMB250102|FRA|2016	LR027524
11	15§	0.11/M	Hospital Cochin (Paris)	Fever, hypotonia	Blood	2016 Sep 5	30	11|COC259064|FRA|2016	LR027525
12	16¶	0.03/M	Hospital (Toulouse)	Acute meningitis, HFMD	Throat swab	2016 Sep 7	33	12|TOU307023|FRA|2016	LR027526
13	17§	1.37/M	Ambulatory (Toulouse)	HFMD	Mouth swab	2016 Sep 14	29	13|PMB263109|FRA|2016	LR027534
14	18§	0.18/F	Hospital Cochin (Paris)	Fever	Feces	2016 Sep 27	20	14|COC286037|FRA|2016	LR027533
15	19§	1.77/F	Daycare (Volvic)	None reported	Feces	2016 Oct 4	29	15|VSV286030|FRA|2016	LR027530
16	20§	1.39/M	Daycare (Volvic)	None reported	Feces	2016 Oct 4	31	16|VSV286032|FRA|2016	LR027536
17	21§	1.69/M	Daycare (Volvic)	None reported	Feces	2016 Oct 4	31	17|VSV286034|FRA|2016	LR027537
18	22§	2.38/F	Daycare (Volvic)	None reported	Feces	2016 Oct 4	29	18|VSV286035|FRA|2016	LR027535
19	23§	2.05/M	Daycare (Volvic)	None reported	Feces	2016 Oct 4	31	19|VSV286036|FRA|2016	LR027538
20	24	0.08/M	Hospital (Toulouse)	Sepsis-like disease	Throat swab	2016 Oct 5	33	20|TOU307031|FRA|2016	
21	25	0.04/M	Hospital (Versailles)	Diarrhea	Cerebrospinal fluid	2016 Oct 9	35	21|VER294019|FRA|2016	
22	26	14.7/M	Hospital (Toulouse)	Acute meningitis, cerebellitis	Throat swab	2016 Oct 10	36	22|TOU307034|FRA|2016	
22	27	14.7/M	Hospital (Toulouse)	Acute meningitis, cerebellitis	Feces	2016 Oct 10	30	22|TOU307035|FRA|2016	
23	28§	0.25/F	Hospital (Toulouse)	Fever	Throat swab	2016 Oct 11	27	23|TOU307036|FRA|2016	LR027532
23	29	0.25/F	Hospital (Toulouse)	Fever	Feces	2016 Oct 12	29	23|TOU307037|FRA|2016	
24	30§	5/M	Hospital (Amiens)	Myelitis	Nasopharyngeal swab	2016 Oct 18	30	24|AMI302002|FRA|2016	LR027546
24	31§	5/M	Hospital (Amiens)	Myelitis	Feces	2016 Oct 20	33	24|AMI302001|FRA|2016	LR027531
25	32§	0.92/F	Ambulatory (Montesson)	Atypical HFMD, herpangina	Throat swab	2017 Jul 3	22	25|PMB501259|FRA|2017	LR027539

*C_t, cycle threshold; EV-A71, enterovirus type A71; HFMD, hand, foot and mouth disease; NR, not reported.
†Patients 15–19 were children in the same day care facility, who had no evidence of clinical disease.
‡Virus genomes were not determined in 12 clinical specimens because gene amplifications were unsuccessful.
§Specimens for which the complete viral genomes (including the full 5’ and 3’ untranslated regions) were determined.
¶Specimens for which partial genomes were determined.

Table 2. Genomes of virus isolates determined from cell culture supernatants to investigate their genetic relationships with the EV-A71 C1v2015*

Sample no.	Sample designation	Collection year	Clinical specimen	Country (city)	Enterovirus type | subgenogroup	GenBank accession nos., earlier partial sequence data	GenBank accession no., this study
1	CF097017|FRA|2003	2003	Stool	France (Clermont-Ferrand)	EV-A71 | C1	HG934182.1	LR027544.1
2	STU562356|DEU|2003	2003	Unknown	Germany (Stuttgart)	EV-A71 | C1	HG934192.1	LR027542.1
3	1480|NLD|2005	2005	Unknown	Netherlands (Eindhoven)	EV-A71 | C1	AB524174.1	LR027541.1
4	CF210042|FRA|2006	2006	Pharynx	France (Clermont-Ferrand)	EV-A71 | C1	HG934219.1	LR027547.1
5	GRE29|FRA|2007	2007	Pharynx	France (Grenoble)	EV-A71 | C1	HG934229.1	LR027545.1
6	STU546711|DEU|2008	2008	Stool	Germany (Stuttgart)	EV-A71 | C1	HG934246.1	LR027543.1
7	37183|TKM|2010	2010	Stool	Turkmenistan	CV-A7	KC879521.1, KC879366.1, KC879445.1	LR027540.1
8	41963|RUS|2011	2011	Stool	Russia	CV-A2	KC879551.1, KC879399.1, KC879478.1	LR027549.1
9	42115|RUS|2011	2011	Stool	Russia	CV-A2	KC879553.1, KC879401.1, KC879480.1	LR027551.1
10	41149|RUS|2011	2011	Stool	Russia	CV-A2	KC879544.1, KC879390.1, KC879469.1	LR027550.1
11	41143|RUS|2011	2011	Stool	Russia	CV-A5	KC879543.1, KC879389.1, KC879468.1	LR027548.1
12	40428|TKM|2011	2011	Stool	Turkmenistan	CV-A6	KC879540.1, KC879386.1, KC879465.1	LR027552.1

*EV-A71, enterovirus A71.

Appendix Table 3. Primers used in this study for gene amplification and amplicon sequencing*

Genomic region†	Primer designation‡	Sequence (5′→3′)	Location§	Use
5′ UTR to 2C	A.F	TAAAACAGCCTGTGGGTTG	2–20	Amplification
	A.R	CATGCAGTTCAAGAGCAARCACCG	4,409–4,432	Amplification
	A.F.1	GACGTCCGGCCCCTGAATGCGGCTAATCC	447–475	Sequencing
	A.R.1	GCGGAACCGACTACTTTGGGTGTCCGGAATTC	536–567	Sequencing
	A.F.2	TGGCTATGGTGAGTGGCC	1,049–1,066	Sequencing
	A.R.2	GCTAGTGACGAGAGTATG	2,610–2,627	Sequencing
	A.F.3	GTCAGATCCCCCAGCACAGG	2,987–3,006	Sequencing
	A.R.3	GTCAGATCCCCCAGCACAGG	2,987–3,006	Sequencing
	A.F.4	GGATACCTCGCCCGATGCGC	3,220–3,239	Sequencing
	A.R.4	GCYCAAGGHTGYGACACGATWGCTC	3,468–3,492	Sequencing
	A.F.5	TCATTGATTGGCTCAAGGAG	4,159–4,178	Sequencing
2C to 3′ UTR	B.F	CATGCAGTTCAAGAGCAARCACCG	4,409–4,432	Amplification
	B.R	GTGGGGGTAAATTTGTTATAACCAGAATAGC	7,378–7,408	Amplification
	B.F.1	GTGTGTGGCAAGGCCATTCAG	4,929–4,949	Sequencing
	B.R.1	GTGTGTGGCAAGGCCATTCAG	4,929–4,949	Sequencing
	B.R.2	GTGATCAACACAGAGCACATGCC	5,694–5,716	Sequencing
	B.F.2	CCRACCCGCACTAARCTTGARCCCAG	5,994–6,019	Sequencing
	B.F.3	GGCCTTGACCTYCCYTACTCYAC	6,381–6,403	Sequencing
	B.R.3	GGCCTTGACCTYCCYTACTCYAC	6,381–6,403	Sequencing
	B.R.4	GGAGYAARYTACCRATYCTACTCCCAGGATCGCTC	6,589–6,623	Sequencing
	B.F.4	CGGTGGACTAAGGACGCACGCAAC	7,143–7,166	Sequencing

*UTR, untranslated region.
†A schematic of gene organization of the enterovirus genome is shown in Figure 2 (main text).
‡A.F and A.R: primers used for gene amplification of the 5′ UTR-2C region. A.F.1–5, forward primers and A.R.1–4, reverse primers used for sequencing the 5′ UTR-2C amplicon. B.F and B.R: primers used for amplification of the 2C-3′ UTR region. B.F.1–4, forward primers and B.R.1–4, reverse primers used for sequencing the 2C-3′ UTR amplicon.
§The location of primers indicates nucleotide positions in the genome of the enterovirus A71 BrCr prototype strain (GenBank accession no. U22521.1).

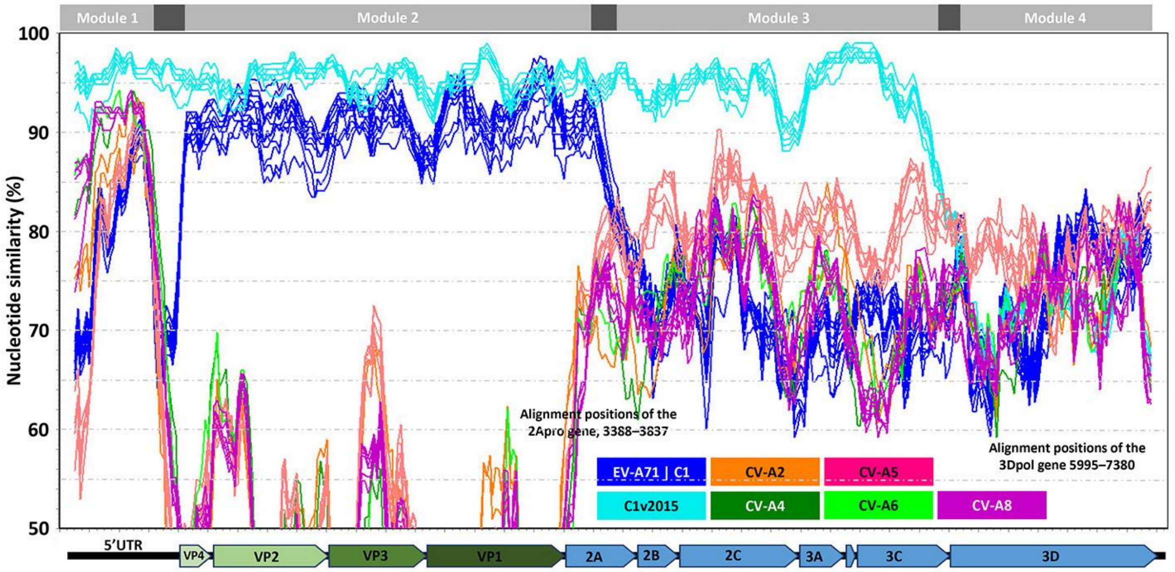

Appendix Figure 1. Nucleotide similarity plots determined for the EV-A71 C1v2015 genome 14|COC286037|FRA|2016 as query (patient 14, infant fever, stool specimen, collection year 2016). The nucleotide similarity was calculated by the sliding window method (window of 200 nucleotides moving every 20 nucleotides). The similarity plots determined for the other C1v2015 genomes were similar. The different enterovirus types and lineages selected for the analysis are color-coded. The mean genetic distance between the C1v2015 and C1 viruses, calculated with the P1 sequences, was <9% nucleotide differences. A schematic diagram of the enterovirus genome is shown at the bottom of the figure. Four genomic modules with different genetic origins were identified as indicated. The 99% confidence intervals (CIs) of the nucleotide boundaries assessed for the genomic modules (indicated in dark gray) were determined as described in Hassel et al. (*1*). The 3′ boundary of module 1 and the 5′ end of module 2 were located within a segment at the end of the 5′ UTR but were not determined precisely. The 3′ boundary of module 2 was located between alignment positions 3,532 and 3,722. The 5′ boundary of module 4 was assessed at the end of 3Cpro gene (alignment positions 5,968–6,044). EV-A71, enterovirus A71; UTR, untranslated region.

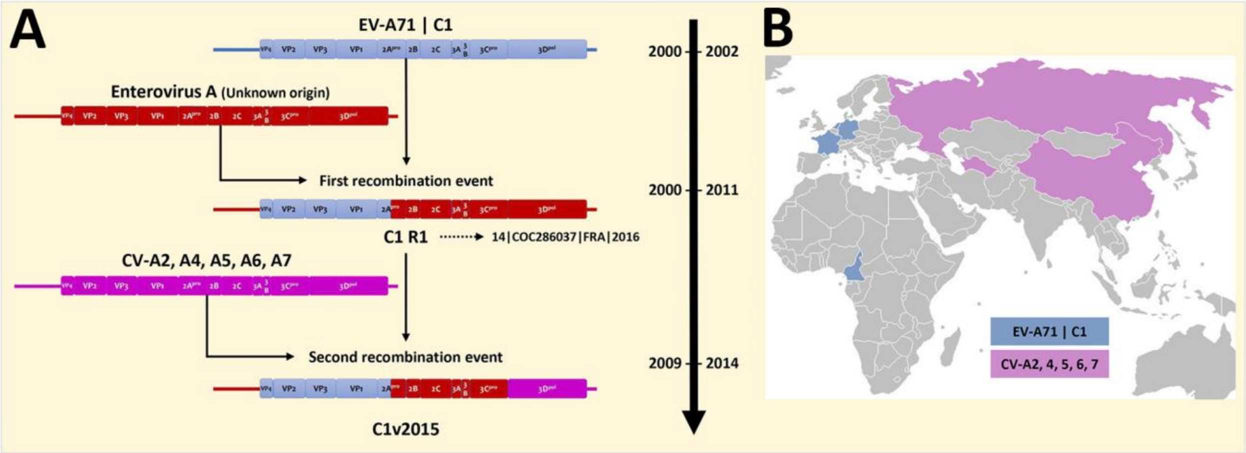

Appendix Figure 2. Scenario of the possible origin and evolution of EV-A71 C1v2015. A) Sequential recombination events that drove emergence of C1v2015. B) Geographic distribution of current gene pools from which the C1v2015 genome was derived: capsid protein genes, countries colored in blue; 3Dpol gene, countries colored in purple. CV-A, coxsackievirus A; EV-A71, enterovirus A71; VP, viral protein.
